# Analysis for policy to overcome barriers to reducing the prevalence of vitamin a deficiency among children (15–23 months) in Iran

**DOI:** 10.1186/s12889-021-11277-8

**Published:** 2021-06-26

**Authors:** Golnaz Rajaeieh, Amirhossein Takian, Naser Kalantari, Fatemeh Mohammadi-Nasrabadi

**Affiliations:** 1 Department of community Nutrition, Faculty of Nutrition Sciences and Food Technology, Shahid Beheshti University of Medical Sciences, Tehran, Iran; 2 Department of Global Health and Public Policy, School of Public Health, Tehran University of Medical Sciences (TUMS), Tehran, Iran; 3 Department of Health Management, Policy & Economics, School of Public Health, Tehran University of Medical Sciences (TUMS), Tehran, Iran; 4 Health Equity Research Centre (HERC), Tehran University of Medical Sciences (TUMS), Tehran, Iran; 5 Food and Nutrition Policy and Planning Research Department, National Nutrition and Food Technology Research Institute (NNFTRI), Faculty of Nutrition Sciences and Food Technology, Shahid Beheshti University of Medical Sciences (SBMU), Tehran, Iran

**Keywords:** Policy analysis, Vitamin a deficiency, Children, Iran

## Abstract

**Background:**

About 30% of children < 5 years old are estimated to experience vitamin A deficiency worldwide. Globally, vitamin A deficiency can be reduced by five major interventions: supplementation, dietary modification, fortification, promotion of both public health, and breastfeeding. This prospective policy analysis (Prospective policy analysis focuses on the future outcomes of a proposed policy. Adapted from Patton, CV, and Sawicki DS. Basic Methods of Policy Analysis and Planning, Prentice-Hall, Inc. New Jersey,1993). (Patton A, Carl V, and David S. Basic methods of policy analysis and planning, prentice-hall, 3th ed. 2012) aimed to identify evidence-based policy options to minimize prevalence (VAD) among 15–23 months-children in Iran.

**Methods:**

Thirty-eight semi-structured face-to-face interviews were held with experts at high, middle, and low managerial levels in Iran’s health system, as well as at Schools of Nutrition Sciences and dietetics, using purposive and snowball sampling. All interviews were recorded by a digital voice recorder and then transcribed, codified, and eventually analyzed using a mixed approach (inductive-deductive) by MAXQDA software version 10.

**Results:**

Most policies related to VAD reduction in this age group are supplementation, expansion of education, and awareness. Three main factors affecting VAD reduction policies emerged from the analysis: basic factors (governance, infrastructure, and organization), underlying factors (social factors, economy), and immediate factors (services). Due to its cross-sectoral nature, evaluating the results of the implementation of this policy requires strong and coherent inter-sectoral cooperation. The existing primary healthcare network (PHC) is a crucial means for successful implementation of policies to address VAD in Iran.

**Conclusions:**

In addition to supplementation and assistance in this age group, other policies should be also planned to reduce VAD in various regions. In addition to the Ministry of Health & Medical Education (MoHME), other actors need to be involved, we advocate, throughout the entire policymaking process of policy-making to reduce VAD in Iran.

**Supplementary Information:**

The online version contains supplementary material available at 10.1186/s12889-021-11277-8.

## Background

Nearly 30% of children under the age of five are estimated to suffer from vitamin A deficiency (VAD) worldwide, and 190 million preschool children every year (2% of all death at this age group) was attributed to VAD [1]. In 2016, 250 million (or 43%) of children living in low- and middle-income countries could not reach their full growth potential and development. Early childhood development (ECD) includes physical, socio-emotional, cognitive, and motor development until age eight [2, 3]. An increased prevalence of VAD was observed among children aged 15–23 months (18.3% in 2012 compared to 2.1% In 2001) in two national surveys [4, 5]. Sistan and Baluchestan province with 20% conjunctival dryness and corneal dryness has the highest vitamin A deficiency among all provinces nationwide. Moreover,, 35% of boys and 21% of girls in the capital city of Tehran had low serum retinol concentrations [6].

VAD is not only is a major cause of death among preschool children but by improving vitamin A levels, several negative health consequences can be avoided. For example, a study has reported a decline as large as 23% in under-five mortality by improving vitamin A levels [7].

Five major interventions are introduced to reduce the prevalence of VAD: (A) - supplementation,(B) dietary modification, (C) fortification, (D) promotion of both public health, and (E) breastfeeding [8].

In 2018, more than 80 countries were implementing universal vitamin A supplementation programs (VASP) specially targeted for children aged 6–59 months by semi-annual national campaigns [9]. A Large-scale cluster-randomized trial conducted in India, titled Deworming and Enhanced Vitamin A Trial (DEVAT), reported that semi-annual VAS were unable to reduce the mortalities caused by VAD [9]. Increased regular intakes of Vitamin A (e.g., through improved diet, fortification, and frequent (daily or weekly) supplements) can reduce the prevalence of VAD [10]. Hence, both long−term (e.g., dietary modification and fortification) and short-term optimal approaches should be initiated in tandem [8] based on the various aspects of countries’ potential solutions and experiences.

The Islamic Republic of Iran, located in west Asia, has diverse geography, culture, and socioeconomic characteristics. The country has been facing epidemiological and demographic transitions that affect its health status [11]. After decades of rapid population growth, ‘Iran’s fertility rate has fallen significantly in recent decades. Iran is experiencing its third epidemiological transition, accompanied by declined rates of mortalities caused by infectious diseases an increased prevalence of non-communicable diseases [12].

The two national population-based surveys conducted in 2001 [4] and 2012 [5] revealed the increasing prevalence of vitamin A deficiency (from 2.1 to 18.3%) in children aged 15 to 23 months in Iran, which indicates the necessity of policy adaptation, with the main focus of identifying evidence-based policy options to reduce the prevalence of VAD among children aged 15–23 months in Iran.

## Methods

This is a qualitative study. We conducted a prospective policy analysis to identify the best options to reduce VAD prevalence among Iranian children aged 15–23 months. Prospective policy analysis focuses on the future outcomes of a proposed policy [13]. Data were collected using semi-structured interviews. Policy triangle framework and stage heuristics were used as conceptual frameworks [14] .Content analysis was used to extract and analyze the themes. We used the purposeful sampling technique to identify 38 interviewees based on their experience and position. Then, using the snowball sampling technique, they were asked to introduce other key informants and experts. Interviewees were policymaker, healthcare manager, expert and academician in nutritional sciences and dietetics, nutrition expert working in comprehensive community health centers, nutrition-related Nongovernmental Organizations (NGO) (e.g., Imam Khomeini Relief Foundation (IKRF); and high-level officials of international agencies, such as United Nations International Children’s Emergency Fund (UNICEF)). The interviewees’ characteristics (*n* = 38) are described in Table 1. An Interview guide was specifically developed based on literature review and “expert’s opinions in this study (Additional file 1). Before interviewing, the interviewer described the objectives and the methodology of the study to all interviewees. Besides, they were assured about the confidentiality of the data. The interviewer guided the semi-structured interviews based on study’s objectives and did not have any assumptions for this policy in Iran. Still, she had a history of a scoping review about policies intended to reduce VAD worldwide. The characteristics of the Research teams are described in Table 2.

**Table 1:** Characteristics of the interviewees

Organization	Code	Position	Age (y)	Education	Number
Ministry of Health and Medical Education (MOHME)	6 2	Head of Department Expert	50–60 40–50	Specialist BSc.	1 2
Vice Chancellor for Public Health (Universities)	1 2 5 9	Vice Chancellor Expert Health care Nutritionist	40–50 30–50 40–50 20–30	Specialist MSc, BS BS,A.S MSc	5 9 5 1
Schools of Nutrition Sciences and Dietetics (Universities of Medical Sciences)	3 4	Dean of school Faculty members	50–60 50–60	**Ph.D.** **Ph.D.**	2 2
Nutrition and food experts and academics	4	Faculty member	40–60	**Ph.D.**	2
NGOs	7 10	Chairman	60–70 50–60	Specialist, **Ph.D.** Specialist	3 1
UNICEF (United Nations International Children’s Emergency Fund)	2	Expert	30–40	**Ph.D.**	1
Imam Khomeini Relief Foundation (IKRF)	2	Expert	40–50	B.Sc	1
Ministry of Interior (Municipalities, Governorates)	8 2	Vice Head Expert	30–60 40–50	**Ph.D.** BSc. Eng.	2 1

**Table 2:** Characteristics of the researchers

	GR	AT	NK	FM
Researcher’s credentials	Msc	MD MPH PhD	MD- Pediatrician	PhD
Researcher’s occupation at the time of the study	Ph.D. Candidate	Professor, Head of Department	Associate Professor, Head of Department	Associate Professor
Male or female	female	male	male	female
Conducted the interview or focus group	Yes	–	–	–
Experience or training	Food policy	Health policy	Food policy	Food policy
male or female	female	male	male	female
Relationship established prior to study commencement	Yes	Yes	Yes	Yes

All interviews were conducted face-to-face at the interviewee’s workplace. Interviews were recorded by a digital voice recorder and then transcribed, codified, and eventually analyzed using a mixed approach (inductive-deductive) by MAXQDA software version 10. On average, interviews lasted 30 min. Interviews continued to the point of saturation. In total, 38 interviews were conducted. Two interviewees refused to participate due to their busy schedules.

Further data were collected through field notes. Transcripts were returned to participants for comment or correction and the research team provided feedback to the participants on the findings. After interpreting the content in each category, the main themes were extracted, all texts were coded, then themes and sub-themes were extracted.

### Quality assurance methods

To assure validity and reliability, coding was performed by two independent researchers and analyzed based on “consolidated criteria for reporting qualitative research (COREQ).

## Results

Most policies related to VAD reduction in Iran emphasize supplementation, expansion of education, and awareness (Table 3). Yet, less attention is paid to planning at different levels for other policies. One interviewee said, *‘unfortunately, they focus only on supplements-related policy; therefore, if you can improve dietary patterns, you might be able to change that policy. Nonetheless, it does not work well, and the strategy was not implemented. I think we should prioritize community education, with emphasis on mothers* (DDPHU2).

**Table 3:** Major Policies related to reduce the prevalence of VAD in Iran (Adapted from ref. No. [15])

Groups of Policy	Policies	Issues
**Direct**	Supplementation	
	Diet modification	Food availability education
	Fortification	Mostly oil
	Agriculture	Agricultural policy, soil enrichment
**Indirect**	Health Policies	Breastfeeding and control of micronutrients
		Improving the organizational capacity of the PHC^a^ network
	Cultural	Food culture

^a^*PHC* Primary health care

Initially, from 1202 codes three main factors affecting VAD policies in this age group were extracted: “basic factors”“, “underlying factors”, and” “immediate factors” Six subthemes and 16 issues were also extracted (Table 4).

**Table 4:** The identified themes and sub-themes

***#***	***Theme***	***Subtheme***	***Issues***
***1***	***Basic factors***	***Governance***	***Evaluation and monitoring***
		***Infrastructure***	***Healthcare network***
			***Registration system***
		***Organization***	***Intersectional cooperation research***
***2***	***Underlying factors***	***Economic factors***	***Budget*** ***Affordability***
		***Social factors***	***Ethnic Composition,*** ***Marginalization,*** ***Food Culture,*** ***Health Demands***
		***Geographical factors***	
***3***	***Immediate factors***	***Policy Implementation***	***Supplementation*** ***Diet,*** ***Health intervention***

### Theme 1: Basic factors

It refers to the essential factors associated with policies intended to reduce the prevalence of VAD. The findings were summarized into three subthemes: governance, infrastructure, and organization.

#### Governance

The MoHME, the main steward of this policy, has several offices and departments, which perform different tasks, and are mostly engaged in policymaking, evaluation, and intersectoral collaboration. Schools of Nutrition Sciences and Dietetics (affiliated to Medical Universities) are the other important actor. An interviewee noted that: *‘Researchers should conduct studies in this arena. All approaches should be investigated, and pros and cons should be reported to policymakers. Then, the strategy with the highest benefits and lowest limitations is proposed”. There are other stakeholders, such as the ministry of agricultural affairs and the ministry of interior affairs., the Islamic Republic of Iran Broadcasting Organization, as a public media, has a crucial role in increasing society’s awareness. Imam Khomeini Relief Foundation is another public organization that has operational responsibilities. (MDd8).*

As we mentioned, various actors were involved in this policy, mainly the MOHME, while the role of other ministries should not be undermined.

##### The ignorance of some stakeholders and politicians about the importance of this policy

There are three problems to set an agenda for the VAD: (1) lack of routine monitoring; (2) VAD is a priority only during crises; (3) in Iran, VAD is prevalent in certain groups and specific regions. An interviewee stated, *‘comparing this to Vitamin D; everyone is interested in this type of vitamin deficiency since years ago, so training sessions are continuously held to improve the situation while VAD is always ignored’* (P9). The most important national research conducted in this field is the National Integrated Micronutrient Survey 2012 (PURA^-^2). However, its conclusions are controversial: *‘Many scholars have doubted the results of Pura 2. After identifying any problem, its causes should be identified; then, all solutions should be identified, and, eventually, decisions should be made regarding the context. In this line, NGOs can have an active role in assisting policymakers (MoHME 2).*

##### Continuous attention to politics

Implementing this policy and prioritizing the importance of this policy depends on the ideas and opinions of the managers of the time.“ *Unfortunately, with the change of managers, the follow-up and implementation of this policy changes, so there is no continuous attention on this policy”(DDPHU2).*

##### Making policy based on evidence

Evidence-based decisions are essential for policymakers; nonetheless, there is a gap due to five reasons: 1) Lack of sufficient research in this area, 2) lack of analytical research design, 3) inability to monitor progress, 4) lack of measurement methods and tools in research, and 5) deficiency in the information. One interviewee stated, *‘They have no data to analyze, which was not considered in the project design. Pura 1 program was executed. Afterwards, they did have unanswered questions during evaluation. Surprisingly, Pura 2 was designed and implemented in the same design. Therefore, the same problems will pop up once again’* (*MoHME* 2).

##### Evaluation and monitoring

Currently, evaluation and monitoring of policies are ignored tools. Hence, data on success rate are ambiguous as the sporadic data on PURA 2 is performed every to ten years. One interviewee noted, *‘The main problem embedded in many national policies is the unavailability of data to make decisions or to assess the effectiveness of ‘policies’* (*MoHME* 2).

Another interviewee said, *‘We see the result in anthropometric indices. We do not have the opportunity for in-depth analysis or follow the case to see what is going ‘on’* (P9). The Deputies for Public Health of Medical Universities perform field visits, which are not systematic, and results do not report to the MoHME.

One interviewee stated, *‘We need to evaluate the outcomes, so we recommend when to start and explain which supplement to use. The networks check whether it is distributed or ‘not’* (DDPHU1). Another participant said, *‘we have a checklist to ask mothers whether they gave the child the supplement. The dose? Since then, we cannot generalize because we want to observe the results in the whole ‘community’* (DDPHU).

Health interventions can be undertaken based on policy evaluation encompassing estimation of needed supplements, registered inhabitants in the systems, verification of registration method, and provision of supplements. One interviewee said, *‘No registration means lack of supplements demands estimation. Seriously, leakage of these medicines to the private sector may cost the person his/her job … 10–15 employees have been fired* “(*MoHME* 2).

A comprehensive evaluation of training is also essential. One participant said, *‘evaluation of training by monitoring and follow-up is important. Nonetheless, since measures are not available, and there are many policies, evaluation is ‘difficult’* (DDPHU2).

#### Infrastructure

The findings related to this subtheme are described in the following.

##### Health networks

One of the Iranian health system’s strengths is its extensive primary healthcare (PHC) network, particularly in rural areas. Through home health centers, people have access to healthcare services. One interviewee stated, *“Our health system has a highly dynamic network in approaching people everywhere and at any time to keep them updated about any emerging issue, which in turn helps implement the related policies such as education and supplementary* ‘*assistance*’ (SNSD4).

*According to the tasks undertaken by the* Iranian health system, the responsibilities of health care providers are continuously increasing while enough human resources are not available. Thereby, services such as education and promotion, particularly in low-income provinces, are not well-performed. One interviewee said, *‘Of course, health care providers are suffering too much workload, which necessarily affects the quality of ‘services’* (SNSD).

Al, low wages and salaries have also led to the dissatisfaction of Behvarzes.^1^ Another participant mentioned, *‘The volume of services provided in our facilities is large. They are included in our service package, children, mothers, middle-aged, elderly, adolescents, and others. Healthcare workers complain about time constraints and inadequate salaries, which demotivate them to carry out core ‘tasks’* (DDPH2).

##### SIB: integrated health portal for Iranians performance

The registration system is new and is not well-developed, and, therefore, is not efficient. That’s why accurate statistics are not available for estimation required for some policies, such as supplementation. One interviewee said, *‘Estimations regarding the supplementations should be based on the data extracted from the information system (*e.g.*, SIB information system). As well, we were faced with challenges related to information collected through information systems; mainly because SIB was not well-prepared, and the required information was not available. Moreover, some families are not registered in information systems, which is an important challenge (DDPH1).*

Another point to consider is differences in data recording systems. SIB is the in-use registration system in all provinces, except Golestan and Khorasan Razavi, in which Nab and Sina systems are currently operated.

This difference might lead to inefficient information exchange. One interviewee mentioned, *‘Since that our provincial system is Nab, the officials in Tehran, for example, cannot see our registered statistics. Why not have a common system? Yesterday, one colleague said: I cannot share information with another Behvarz in another province due to differences in the registration ‘system’* (DDPH1).

##### Lack of suitable working conditions for health workers

Despite the increasing duties of health workers, their salary is insufficient and their number is low.” “*Certainly, we have a lot of trouble discussing training. Why?” “Meditation assignments are so heavy that he may not have the opportunity at all, and there are so many referrals that he has so many assignments*.” (DDPH5).

### Organization

#### Intersectoral cooperation

Policies related to reducing VAD require both inter-sectoral and intra-sectoral collaborations. Inconsistency stemming from lacking synchronization between administrative and academic systems, fragmented working units, and poor coordination with doctors (especially non-faculty members) might adversely affect this policy.

One respondent said, *‘Unfortunately, you see a lot of disparity between specialist levels and health levels throughout the country. Experts claim there are high expectations of the health system; meanwhile, doctors claim that patients are given whatever they need according to available services. Yet, many items are missed, such as commercial ‘medicines’* (SNSD4).

Lack of collaboration can be due to physicians’ insufficient training, focusing on curative interventions (instead of preventive ones), insufficient quality of supplements, wrong attitudes, and lack of physicians’ involvement in developing processes.

Another interviewee mentioned, *‘Physicians are more clinically-oriented on treatment but not prevention. Indeed, doctors’ orientation in the past was towards prevention; however, the modernized higher education system has sacrificed those well-oriented ‘doctors’* (*MoHME* 6).

### Theme 2: Underlying factors

This theme refers to contextual factors, which have been summarized under three subthemes: economic, societal, and geographical factors.

#### Economic factors

In this section, two main factors were found: budget and affordability (price).

##### Budget

The budget stands for the amount of money required to implement policy and funds allocated to medical universities for executing this policy. Some provinces don’t spend this budget in so doing. One interviewee said, *‘Incidentally, Sistan and Baluchistan provide some budget information, but not the whole budget is gone for implementing the policy”* (DDPH2).

##### Affordability

One of the causes accounting for policy failure is the hard economic situation resulting from the imposed sanctions and increase food prices. These supplements and foodstuffs needed for implementing the policy are unaffordable. One participant said, *‘Not only educating people is important, but also to which extent requirements are affordable to ‘them’* (NGO10).

##### Women’s employment

In some families, women are forced to work. This issue may face many challenges in caring for their children, which adversely affects their diet, growth, and health status. One interviewee mentioned, *‘In some families, mothers work in paddy to struggle against poverty. Meanwhile, their elder children care for younger siblings. It is a real ‘problem’* (DDPH5).

Working women do not have the opportunity to take care of their children. Mothers of children who go to paddy farming cannot take care of their children and are usually left to older children and cannot take good care of them—(DDPH10).

#### Societal factors

NGOs can play a paramount role in more than one field, such as education, agenda-setting, and financing. However, for the moment, NGOs dedicate their efforts to theoretical and clinical-oriented education. One interviewee said, *‘certainly, it depends on how we benefit from their field of interest. NGOs have their scope and aims; meanwhile, engaging them in various activities and planning for future policies will reinforce their role in helping the national health system achieve its ‘goals’* (1). Another interviewee said, *‘Unfortunately, our NGOs these days pay more attention to treatment and clinical guidelines rather than preventive care. Hence, I don’t know any of the current employees in ‘NGOs’* (*MoHME* 6).

The food and pharmaceutical industry in the private sector, can also take part in improving food, supplements, and quality of medicines. Moreover, promoting the food and pharmaceutical industry can improve food, supplements, and quality of medicines, e.g. promoting and advertising vitamins. Consequently, careful planning and policy oversight is required.

Health volunteers can also train and motivate people to improve the condition. “Unfortunately, this resource is not used at the moment and is not very useful.” (DDPH9).

##### Ethnic composition

Due to food insecurity, many people from Sistan and Baluchistan provinces have migrated to other provinces like Golestan; thereby, VAD became more prevalent. One interviewee mentioned, *‘Which groups in Golestan have more VAD? The answer is those migrants from Sistan and Baluchistan. They live in all parts of the ‘province’* (DDPH2).

##### Marginalization

Migration to find a job, etc., causes more severe illness, and people may live in places that do not have access to appropriate services.

‘From provincial capitals to suburbs or remote areas, living conditions are changing. Accordingly, fair attention should be ‘paid’ (DDPH1).

Inhabitants in the outskirts may experience economic and social problems due to the low quality and quantity of provided services. Health problems such as VAD may arise in such circumstances. One participant said, *‘People in outskirts are mostly unemployed poor migrants. Policymakers have to find effective solutions for those people through the health ‘system’* (DDPH2).

##### Food culture

This section describes cultural food habits in specific areas. Generally, consumption, e.g., fruits and vegetables, is less than recommended due to various reasons. According to the interviewees, public awareness about healthy food has been promoted because of media efforts, but this should be translated into tangible outcomes.

*“Most Turkmen do not consider vegetables as food and consume very little” (NFEA4).*

##### Health demands

Lack of public accountability may lead to adverse implications. For example, some people give up taking multi-dose vaccines or supplements. One interviewee said, *‘We use SIB registration system, which detected that around 30% of people did not receive their ‘supplements’* (SNSD3).

Ordinary people tend to use traditional prescriptions regardless of their safety and efficacy. One participant stated, *‘New prescriptions are always interesting for laypersons. Hence, many of them tend to use traditional prescriptions without verifying their ‘contents’* (DDPH5).

#### Geographical factors

Access to food resources and even health services is limited due to geographical barriers in some places in the country” Iran has different geographical areas, so access to resources can be different” (MI8).

### Theme 3: Immediate factors

This theme is related to tangible and early return factors to reducing the prevalence of VAD. It embraces one subtheme: Policy Implementation*,* which can be explained through three subtopics (Major policies in the Iran) of supplementation, diet, and health interventions.

#### Policy implementation in the country

##### Supplementation

Supplementation is the most common policy for VA-deficient children in Iran. It is free for children up to 6 months as they take vitamins A + D, then multivitamins. Yet, there are some challenges in this regard. One interviewee mentioned *‘Vitamin A drops are the most effective and direct intervention in our country. Hopefully, the policy related to micronutrients and supplementation will be ‘implemented’* (*MoHME* 6).

As a short-term solution and low prices, supplements are the most desirable policy for both government and people. One interviewee said, *‘I believe that these supplementations should always be considered a short-term ‘solution’* (*MoHME* 2). Another interviewee stated *‘supplements, as drops dosage form, are less expensive and affordable for most families* (P9). In some urgent conditions such as floods, mega-dose is recommended as a compensatory therapy. An interviewee said, *‘in the recent five years, the demand for mega-dose therapy has risen, especially in Lorestan, Khuzestan, and Golestan provinces in which floods and similar crises are common.* (*MoHME* 6).

Dose interruption is also an issue; some mothers do not continue the dosing schedule, particularly when there is no vaccination, while others show their carelessness. One interviewee said, *‘It is preferred to give mothers supplements enough for three months, with follow up by phone or SMS as a reminder, or even short ‘visits’* (DDPH1).

Shortage in the budget is another problem. An interviewee said, *‘By the recent transformational plan, we try to cover the whole need for children’s supplements. Meanwhile, no budget is available for so ‘doing’* (DDPH1). Another interviewee stated, *‘We attempt to prioritize rural and remote areas as they face more challenges in getting supplements for their children. So, we recommend some people to buy while introducing free medicines to ‘others’* (DDPH1).

##### Diet

The MoHME endeavors to provide food packages to children of needy families collaboratively with the Imam Khomeini Relief Committee in each province. One interviewee said, *‘The MoHME has initiated some policies to introduce food packages to some needy families. s’*(IKRF2). Sometimes, these packages do not involve enough micronutrients and are not limited to target groups. One participant said, *‘In deprived provinces, the packages almost lack fruits and vegetables, and micro-nutrients. We have reported to the MoHME, but no response was given so ‘far’* (SNSD4).

Insufficient cooperation between actors results in a weakness in this policy. One interviewee said, *‘Right now, the MOHME cooperation with other ministries is ‘questionable’* (DDPH1). Raising awareness, continuous education, effective training are the main pillars in achieving policy goals. One interviewee said, *‘Workshops are held to educate attendees on how to modify their food style. These activities are good, but the target group should be different in each ‘session’* (DDPH1). Volunteers can play an important role when taking part in such programs. One participant said *‘Interested volunteers were selected through interviews, then trained, afterward, they embark on educating and training others in mosques or even* via *Telegram ‘channels’* (DDPH2). Interviewees stated some other obstacles, including; time limitation for training, lack of response to traditional teaching methods, and improper policy implementation.

Home gardens are also one of the policies that should be done in cooperation with the Ministry of Jihad for Agriculture. This policy is not possible due to the facilities that exist and the geographical differences in all areas.

*Successful implementations of home gardens in provinces such as Sistan and Baluchestan due to their geographical conditions require special arrangements with the cooperation of Jihad Agriculture “(DDPH2).*

##### Health interventions

One of the health interventions in Iran is breastfeeding. One interviewee said, ‘*We did a very good job of breastfeeding training. Every mother should immediately go to a health center for screening tests directly after birth. In the meantime, breastfeeding is especially important. We even know how many mothers use breast milk or formula ‘milk’* (DDPH5).

Another interviewee stated, ‘*All the instructions that we give are integrated into two programs: the integrated care of a healthy and sick child. The Comprehensive Breastfeeding Program is complementary to these supplementary ‘guidelines’* (*MoHME2*).

## Discussion

The present study aimed to analyze the policy to reduce VAD prevalence among children aged 15–23 months in Iran.

### Summary of main findings

In this study, factors influencing the established policies were grouped into three categories; basic, underlying, and immediate. Each category is subdivided into subcategories which can contribute to evidence-based policy. Currently, the main policy in this specific age group in Iran is supplementation. Obstacles to the successful implementation of this policy include inaccurate evaluation, incomplete information and statistics, lack of clarity of the importance of this policy for policymakers, inconsistencies in actors’ performance, economic problems, and some social and cultural characteristics such as demanding health. Factors affecting dietary changes such as training or home gardens have been more or less considered, but there are many problems in their implementation.

### Relevance of findings

Before a policy can be launched, the “government’s attention should be drawn to the underlying issue [16]. Our interviews confirmed that VAD prevalence in this age group is not clear to politicians; additionally, the existing policy is neither carried out constantly nor with strict supervision, but sometimes it is changed before coming to its end. Sustainable programs require political commitment regardless of any related changes [13]. The implementation of this policy depends on ‘politicians’ perspectives in each province. Politics is done at different levels, and in several cases, budgets are spent on some other policies. Therefore, policy importance should be articulated with actors such as civil society and IRIB on politicians.

There are two national studies on micronutrients in Iran, but their results have been considered to make decisions to reduce VAD prevalence. The criteria for moderate and severe VAD in the study of 2001 were less than 30 and 20 μg/dL, respectively, which were modified to less than 20 and 10 μg/dL in 2012, based on the updated WHO guidelines, respectively. Despite this change in the criteria, there is an increase in the prevalence of VAD in this age group [4, 5] which can be partially attributed to deteriorate the economic status of Iranian population. Based on WHO classification, VAD in these two study groups was moderate (≥10% to ≤20%) [17]. Evidence-based decision-making has been proffered for explicitly justified decisions [18, 19], so it is expected that physicians, as well as politicians to rely on authentic evidence when making decisions or even policies [20]. The main purpose for so doing is to grasp the three streams together, i.e., problem, policy, politics, to develop the content based on specific policy elements which are likely to be effective, then decide on the next step; improve, expand, or terminate that policy [21]. Schools of nutrition and scientific associations can better cooperate to find out these evidences. Scientific studies should be planned analytically rather than being descriptive.

It seems that the relevance of the knowledge provided by public health actors is not limited to the agenda-setting stage [22]. There are many components to developing and implementing policies that should be monitored and evaluated intermittently to determine if interventions are necessary [23]. The evaluation aimed to determine the relevance and consistency with objectives, developmental efficiency, effectiveness, impact, and sustainability [24]. Termination of policies might be attributed to inefficiency or lack of stakeholders and elected officials who first put it on their agenda [16].

For accurate planning and evaluation, a reliable information system is required, as the information may differ for each stage of policymaking. Accordingly, the information should be recorded correctly [25]. However, there are some problems such as underdevelopment of the *SIB* system, differences in the registration systems, and time constraints required for recording information. The workload of health providers without sufficient incentives is one of the consequences which might negatively affect policy implementation.

Although the Iranian PHC system has numerous successes, especially in health network deployment, Behavers’ role, health indicator improvement in rural areas, and the elimination of urban-rural inequality [26], he is one of the main strengths implementing related policies such as supplementary foods and promoting education.

The role and importance of context in policy implementation are widely recognized. The main contextual factors that significantly affect the promotion and use of knowledge in policymaking have to be detected and explored [27]. In Iran, there are various ethnic groups, cultures, climates, and social and economic situations. Socioeconomic and cultural factors significantly affect access level to vitamin resources to the population [28]. For success, all stakeholders should be involved in decision-making and implementation processes seeking commitment, ownership, and accountability of government, civil society, combined with advocacy and assistance of international agencies [29]. Therefore, to implement this policy properly, there is a need for inter-sectoral coordination and cooperation. For example, Imam Khomeini’s relief committee is currently distributing food packages to needy families, but these packages need to be evaluated to enrich them in terms of vitamins. The monthly food rations in a province cans consist of vegetable oil (2 L), rice (10 kg), lentil (1 kg), milk (3 L), soybean (0.5 kg), canned tuna (three cans), and spaghetti (2 k). In contrast, in another province it contained milk (2 L), cheese (0.5 kg), potato(3 kg), vegetable oil (2 L), canned tuna (five cans), rice (2 kg), wax bean 1 kg), lentils (1 kg), eggs (1 kg), and chicken (2 kg) [30, 31]. IRIB may also have a great impact on education and cultural factors. MOHME should be responsible for organizing these efforts [30, 31].

Supplementation and training are the major policies to reduce VAD prevalence in Iran. Success in supplementary policy depends on accurate evaluation, supplement coverage, and political commitment [32]. In South Asian countries, VA supplement was a successful approach to overcome VAD, as these programs were well-structured and closely monitored [33]. Effective training should start from the local knowledge in that area [29] and can help with dietary changes. Still, there was no formal and accurate evaluation of this policy. Due to the workload of health providers and time constraints, health care providers cannot evaluate the effectiveness of learning and the amount and use of supplements. Assessment is carried out in the field by inspectors of the university’s health deputy. Therefore, accurate evaluation of the supplementary is not performed in different stages, such as estimating the required supplements, delivery of the supplement to mothers, and the amount for use and did not provide credible and useful information, enabling incorporating lessons learned into the decision-making process of both recipients and donors. It is essential to officially evaluate the outputs of this policy by engaging all interested actors.

The evidence from this study implies there is no enough political commitment and inter-organizational cooperation in this policy. Sometimes decision-making is subjective to prejudgments, rooted in ‘policymakers’ views, and without a comprehensive understanding of the problem. Based on Bangladesh’s experiences, political commitment is required for policy triumph [29, 34]. In 1997, reducing child mortality became a political priority for the Nigerian government. Ministry of Health, Helen Keller International Foundation, and UNICEF have formed a coalition to control VAD. Since then, Nigeria became one of the first African countries to effectively supplement VA during the National Immunization Days to eradicate polio [35]. Despite this, many countries use the mega-dose vitamin in VA deficient areas (of course, it recommended shifting judiciously from periodic VACs to increasing regular intakes) [10]. Mega-dose has not been provided to children with VAD for a long time. Still, his supplement is given to children up to 6 months and then substituted by multivitamins for free in the amount of vitamin consumed according to the age.

Except for physical access to vitamins, geographical or economic reasons, cultural factors, and common diet type in those areas are also influential factors [36, 37]. Accountability of society and interested people affect this policy, so cultural factors such as lifestyle, context, and marginalization which affect these people, should be deemed. Many children between the ages of 18 and 23 months, who are not vaccinated, do not visit health centers to get supplements or in some cases, not enough supplements are provided in health centers. Either they may take different types of supplements following non-specialists or in doses lower than recommended.

It is recommended that other policies be used along with supplementary to boost VAD reduction [38]. Most VA food sources can be cultivated and accessed in developing countries [39]. In Iran, various diets and dietary resources are very low due to various factors, such as cultural and economic factors. Dietary diversification and ensuring regular access to foods naturally rich in VA are important in the long run [34]. Home gardens provide fresh sources of vitamins and increase women’s participation [40]. Even in areas where water and land are scarce, using innovative approaches to home gardening can be effective for families [41].

One of the important policies to control vitamin deficiency is breastfeeding [29]. Breastfeeding training is given, but the key issue is the mother’s access to VA resources, which depends on various factors and needs that should be considered.

As mentioned, several factors contribute to the proper implementation of the VAD prevention policy, which makes it possible for various organizations such as the Ministry of Interior, the Ministry of Welfare, the Civil Society, and IRIB to cooperate and coordinate with the MoHME. Also, the departments of MoHME, such as Food and Drugs, the Health Deputy, and the Education Deputy, have to be coordinating with each other.

It is noteworthy that affordability and access to policy requirements dominate education and awareness concerning its content; this highlights the role of government, NGOs, and international organizations in aiding needy people in various packages of foods and supplements [42].

#### Limitations/strengths

The current study had limitations. Firstly, we were unable to interview experts in the Ministry of Agriculture and the Islamic Republic of Iran Broadcasting (IRIB). Since the current study had a qualitative framework, the findings have low generalizability. According to the author’s best knowledge, the current research is the first study on the prevalence of VAD in children aged 15–23 months.

### Policy recommendations

Based on our findings, we recommend that each province in Iran will identify facilitators and administrative barriers to address VAD, and through multi-sectoral collaboration, formulate evidence-based policies to tackle the problem. In addition to supplementation policy, other policies should be planned.

In particular, we recommend providing quality training on proper nutrition, supplementation through actors such as nutritionists, caregivers, doctors, media, municipalities and relief committees. In addition, accurate assessments of all stages of policy through various actors, with relevant job description is crucial for recording information and statistics and results. NGOs can also be helpful in providing food packages containing vitamin A sources at reasonable prices, full coverage of supplements in deprived areas, and increasing people’s nutritional literacy. Adopting appropriate economic policies to increase access to vitamin A sources, with special emphasis on low-income and vulnerable groups (e.g., through subsidies and incentive policies for health-supporting industries); improving the working conditions of health workers and employing more healthcare staff; monitoring the allocation of earmarked budget for reducing the prevalence of VAD; and implementing policies related to home gardens to increase growing vegetables that are rich in vitamin A; are among policies that we recommend to be implemented to tackle VAD in Iran. Fortification of infant and children foods with vitamin A is a cost-effective intervention for reducing vitamin A deficiency, especially in settings where improving dietary quality through food variety is not possible.

## Conclusions

WHO has prioritized early child development (ECD) as a window of opportunity to improve both health and equity. Essential micronutrients play a vital role in child development. Providing supplementation is the main feasible intervention to reduce VAD in Iran [15]. Although this policy is necessary, it might be insufficient since several other macro factors, which mostly are out of the health sector’s control, including governance, infrastructure, organization, social factors, and economy, could also contribute to children’s developmental outcomes. To reduce the prevalence of VAD in this age group, revising policies with particular emphasis on vitamins in this specific age group with a precise definition of indicators is crucial. The country should pay special attention to other policies such as education in diet change and food diversity, and home gardens according to specific conditions of each region.

## Supplementary Information

**Additional file 1.** Interview guide.

## Data Availability

The datasets used and / or analysed during the current study are available from the corresponding author upon reasonable request.

## Abbreviations

VAD: Vitamin A deficiency
DEVAT: Deworming and Enhanced Vitamin A Trial
VASP: VA supplementation programs
NGO: Nongovernmental Organizations
IKRF: Imam Khomeini Relief Foundation
PHC: Primary health care
UNICEF: United Nations International Children’s Fund
MoHME: Ministry of Health and Medical Education
DDPH: Dean Deputy for Public Health A specific office in the MOHME will take the principal responsibility for policymaking
IKRF: Imam Khomeini Relief Foundation
P: Pediatrician
SNSD: School of Nutrition Sciences and Dietic
MFE: Nutition and food experts and academic
MI: Ministry of Interior

## Footnotes

1 Health workers in the village health house
