# Supplementary material for: Analysis for policy to overcome barriers to reducing the prevalence of vitamin a deficiency among children (15–23 months) in Iran
Source: BMC Public Health. 2021 Jun 26;21:1234. doi: 10.1186/s12889-021-11277-8 (PMC8236148; doi:10.1186/s12889-021-11277-8)
Supplement: Supplementary file 1 — Additional file 1. Interview guide. [file 12889_2021_11277_MOESM1_ESM.docx]

**Additional files**

Additional file 1: Interview guide

• What are the main policies and these challenges to reduce vitamin A deficiency in Iran?

• What are the documents related to reducing vitamin A deficiency?

• Are these policies implemented completely? Who is involved in the implementation of these policies?

• Do you think that vitamin A supplementation in children aged 15-23 months is enough to reduce vitamin A deficiency? Do you think there is a specific policy that has not yet been implemented?

• What policies do you think should be to consume more rich sources of vitamin A? What are the obstacles in this regard?

• How has the prevalence of malnutrition changed in children aged 15 to 23 months?

• What are the differences between the level of access to food, safe water, literacy, and awareness of women?

• Do you think the dominant diets in Iran provide a sufficient intake of vitamin A?

• How does the implementation of policies reduce vitamin A deficiency in the country?

• Does this policy been successful?

• Is there an evaluation/ monitoring system related to reducing the prevalence of vitamin A policies, such as supplementation, breastfeeding, eating vitamin-rich sources?

• In your opinion, what Facilitators and barriers are there to reduce the prevalence of vitamin A deficiency in the country?

• In your opinion, what are the main key players that can reduce vitamin A deficiency in Iran? What can be done to improve their performance?

• How do you assess the role of the media in reducing the prevalence of vitamin A deficiency in the country? What can we do to Increase media participation?

• How do you assess the role of the private sector in reducing the prevalence of vitamin A deficiency in the country? What can we do to Increase private sector participation?

• How do you assess the role of the civil society in reducing the prevalence of vitamin A deficiency in the country What can we do to Increase civil society participation?

• How do the industrial sector can reduce the prevalence of vitamin A deficiency? What are the problems in this regard?

• Finally, is there another point about reducing vitamin A deficiency that needs to be discussed and not addressed?
